# 

**Published:** 1890-01

**Authors:** 


### CONTENTS—JANUARY, 1890.—VOL. 5, NO. 7.

ORIGINAL COMMUNICATIONS— Page

Milk Inspection in Cities. W. M. Yandell, M. D., Health Officer, El Paso . . . . . . . . . . . . . . . 239

Surgical Clinic, Memphis, Tennessee.—Notes from Hospital Practice. W. B. Rogers, M. D . . . . . . . . . 244

Report of Two Deaths from Partial Chloroform-Anæsthesia, with Remarks. Dr. J. T. Harrington . . . . 248

Tape Worm. Dr. W. A. Rape . . . . . . . . . . . . 251

Sub-Peritoneal Hæmatocele. Dr. Eugene Clarke . . . 252

CORRESPONDENCE—

Gastritis in Children. Dr. J. L. Cunningham . . . . 255

Extra-Uterine Pregnancy. Dr. E. McD. Bridgeford, St. Louis . . . . . . . . . . . . . . . . . . . . . 258

A Second Sarah. Dr. R. A. Taylor . . . . . . . . . 259

SOCIETY NOTES—

Ninth Quarterly Meeting of the Austin District Medical Association . . . . . . . . . . . . . . . . . . . 259

West Texas District Medical Association . . . . . . . 262

Central Texas Medical Society . . . . . . . . . . . 262

EDITORIAL—

Is the Influence of the Medical Profession on the Wane? 263

Similia Similibus? . . . . . . . . . . . . . . . . . 267

Dr. McLaughlin's New Hemostatic Forceps . . . . . 268

Important Announcement. Biography of Contemporary Physicians . . . . . . . . . . . . . . . . . . . 269

MEDICAL NEWS AND MISCELLANY

Numerous Items of News of interest to the Profession . 270

BOOK NOTICES . . . . . . . . . . . . . . . . . . . . 275

PUBLISHER'S NOTES . . . . . . . . . . . . . . . . . 278

